# Editorial: Sustainable and resilient food systems in times of crises

**DOI:** 10.3389/fnut.2025.1564950

**Published:** 2025-02-11

**Authors:** Tarek Ben Hassen, Hamid El Bilali, Bassel Daher, Stefan Burkart

**Affiliations:** ^1^Department of International Affairs, College of Arts and Sciences, Qatar University, Doha, Qatar; ^2^International Centre for Advanced Mediterranean Agronomic Studies (CIHEAM-Bari), Bari, Italy; ^3^Texas A&M Energy Institute, College Station, TX, United States; ^4^Department of Biological and Agricultural Engineering, Texas A&M University, College Station, TX, United States; ^5^International Center for Tropical Agriculture (CIAT), Cali, Colombia

**Keywords:** sustainability, food system, resilience, crises, climate change

The global food system is facing multiple and multifaceted challenges and crises: increasing population, climate change, pandemics, conflicts, economic shocks, and natural disasters. These challenges have exposed its fragility and underscored the urgency of transitioning to sustainable and resilient food systems. Such systems ensure equitable access to nutritious food, minimize environmental impacts, and enhance resilience against shocks. In crises like the COVID-19 pandemic and the ongoing conflict in Ukraine, the vulnerabilities of the global food system have become starkly apparent, leading to disruptions in supply chains, food shortages, and inflation. Additionally, climate change and natural disasters further threaten food security worldwide. This Research Topic explores innovative approaches—including agroecological practices, circular economies, and localized food systems—to build resilience and sustainability. To provide a comprehensive understanding of these pressing issues, the 17 articles of this Research Topic are organized into four thematic areas: the impacts of crises on food systems, the effects of COVID-19 on food systems, sustainable food systems and practices, and the social, economic, and waste management dimensions of food systems.

The fragility of global food systems has been underscored by recent crises, including conflicts, natural disasters, and economic disruptions, which have exposed vulnerabilities in achieving food security and nutrition. For instance, the 2023 war on Gaza has significantly set back progress toward Sustainable Development Goal (SDG) 2 to end hunger. As highlighted by Hassoun et al., the ongoing war on Gaza has escalated acute food insecurity, with relentless bombardment and ground operations destroying agricultural lands, food production infrastructure, and Gaza's entire food system. Facing catastrophic hunger, some families eat animal feed and weeds to survive. This humanitarian disaster threatens global food security and progress on SDG 2. While international organizations strive to alleviate the situation, sustainable solutions addressing root causes are urgently needed to ensure adequate and nutritious food for Gaza's population.

Further, the Lebanese food system has been under stress due to many multiform crises and shocks, which makes it an interesting case study. Makoukji et al. shed light on the evolution of food (in)security status among residents in Karantina (a neighborhood in Beirut) after the explosion in Beirut Port in 2020. They showed a significant drop in food security and diet quality among residents during the 2 years following the explosion, with nearly all the residents turning to crisis coping methods. They highlighted the need for assistance and reforms to restore food systems and enhance resilience in regions affected by crises.

Similarly, the disruption of grain trade caused by geopolitical tensions has threatened food security in the Middle East and North Africa (MENA) region, exacerbating existing vulnerabilities. Rahimi et al. demonstrated that 40% of dietary energy (1,261 kcal/capita/day) and 63% of protein (55 g/capita/day) in the MENA region come from imported grains, including 164 kcal and 11 g of protein from Russia and Ukraine. A complete halt in imports from these two countries would severely challenge food security in the region. This study emphasized the urgent need for proactive measures to ensure a stable food and feed supply.

Analyzing the resilience of food systems in six Asian countries amid the COVID-19 pandemic, geopolitical conflicts, and climate change, Favas et al. identify significant outcomes such as currency depreciation in Sri Lanka, Pakistan, and Lao PDR and widespread food price inflation. While countries with higher resilience capacities reported milder food insecurity impacts, others faced escalating vulnerabilities. Key trends include increased crop production and reduced food imports, with variations in government support. Recommendations emphasize integrating climate adaptation measures, enhancing resilience assessments, and adopting proactive intervention strategies to ensure long-term food system stability.

The COVID-19 pandemic is one of the most critical crises and shocks faced by the global food system, revealing vulnerabilities, and prompting adaptive responses across diverse contexts.

During the pandemic, Italy was the first European country to implement a nationwide lockdown, and schools were closed for a prolonged period. However, Pagliarino revealed that access to school meals was disrupted due to these closures, potentially decreasing student nutrient intake and household food security. Later, when schools were open, the service changed by responding to preventive measures defined at the central government level and implemented locally through coordination between municipalities, catering companies, and schools. Children, teachers, and families, i.e., the service's end users, were excluded from these discussions. The result was a mismatch between pandemic prevention goals and social values in school catering.

Similarly, the pandemic has disrupted the food system in Manitoba, Canada. However, Lowitt et al. confirmed that government measures, including funding for food banks and local food initiatives, helped mitigate disruptions. Nevertheless, food insecurity persisted, especially among vulnerable groups, with a 30% rise in food bank usage and challenges in Northern Indigenous communities. Labor shortages affected the agri-food sector, while interest in local food systems and urban gardening surged. The article emphasizes evaluating pandemic policies, monitoring food security for at-risk populations, and strengthening local food systems to enhance resilience and equity in post-pandemic recovery.

Likewise, the COVID-19 pandemic profoundly impacted food systems in China, highlighting significant challenges. For instance, Han et al. analyzed how the lockdown enacted in Shanghai in 2022 disturbed the city's food system and resulted in cases of food insecurity and the responses of the local government in collaboration with various stakeholders. The solutions suggested aim, inter alia, at stabilizing food supply and prices, upgrading transportation capacity, and improving food access channels with a particular focus on vulnerable groups.

Moreover, Fang et al. investigate the impacts of the COVID-19 pandemic on the achievement of zero hunger in China, with a particular emphasis on improving nutrition. They postulated that the pandemic affected agricultural production and slowed down efforts to eliminate hunger and promote nutritional sustainability. Based on the findings covering 29 provinces in China, they formulated several policy recommendations, such as establishing a robust natural disaster prevention and control system and fostering agricultural technological innovation to mitigate the adverse effects of food security emergencies and strengthen the resilience of food systems in the future.

Further, with the challenges faced by institutional food services during the pandemic, the focus shifts to alternative food networks (AFNs) that emerged as vital mechanisms for resilience and adaptation. In the Czech Republic, the results of Smutná et al. showed that 68% of Czech households engage in AFNs, including gardening and market-based options like farmers' markets and farm gate sales. However, COVID-19 and economic pressures in 2021 led to reduced consumption of organic food (−22%) and fresh produce (−10%) and increased food insecurity (+18%). The article explores the role of AFNs in supporting food and nutritional resilience while addressing challenges that threaten market-oriented AFNs.

Building sustainable food systems is essential for addressing environmental challenges, enhancing resilience, and ensuring equitable access to nutritious food.

In Tunisia, the study of Souissi et al. underlined that farmer organizations play an essential role in advancing sustainable farming through clear goals and collaboration despite challenges like input scarcity, water shortages, low income, and market access. Over 90% of farmers receiving agroecological training reported adopting new practices, with the olive oil value chain showing strong potential for transformation despite constraints like climate and lack of policy support. Key drivers include agroecological practices' compatibility with farmer skills and their environmental and economic benefits. The study emphasizes participatory approaches and socio-technical systems analysis to address barriers, integrate diverse farmer perspectives, and support Tunisia's transition to sustainable, resilient food systems.

In Burkina Faso, Tapsoba et al. underscored the crucial role of the food environment in achieving sustainable food systems. This is due, among others, to the centrality of the food environment in reducing malnutrition in all its forms and combating non-communicable diseases. Against this backdrop, they use the Healthy Food and Environment Policy Index (Food-EPI) methodology with a group of national experts to develop and prioritize actionable recommendations for the Burkinabe government to establish a healthy food environment. They formulated 20 recommendations related to policy (e.g., regulating food advertising/marketing) and infrastructure support (e.g., investments in population nutrition) domains.

Further, Singh et al. explore the indigenous chicken production system (ICPS) in three agro-ecologies of the Indian Himalayan Region, examining its role in food and economic security. Findings reveal that sub-temperate regions have larger flock sizes, higher egg production, and better household dietary diversity than tropical and sub-tropical zones. ICPS contributes 18% of household income in sub-temperate areas despite challenges like disease, predation, and limited chicken availability. The system enhances ecological resilience through farmyard manure use but requires targeted health and management interventions to boost productivity.

Finally, Maulu et al. examine the role of fish in addressing food and nutrition security challenges across 10 Southern African countries, including Angola, Zambia, and South Africa. Persistent issues such as overfishing, climate shocks, and limited aquaculture investment hinder fish production, while inadequate access to quality inputs further exacerbates malnutrition. Opportunities exist to promote sustainable fisheries and aquaculture, especially in landlocked nations exploring aquaculture solutions. Countries with extensive coastlines show potential for fishery development. Addressing these challenges requires governance reforms, technological innovation, policy support, and investment to enhance sustainability and resilience in the region's fisheries and aquaculture industries.

Understanding the social, economic, and waste management dimensions of food systems is vital for fostering equity, reducing waste, and enhancing resilience in the face of crises.

In Italy, Catalano et al. examined food waste awareness among university students, highlighting key attitudes, behaviors, and opportunities for promoting sustainable consumption practices. The survey reveals high engagement in sustainable food purchasing but low adherence to waste prevention practices at home. Key factors influencing food waste include lack of involvement in grocery shopping and male gender. Among subgroups, only those with structured nutrition education handled “use by” products significantly better than peers without such training. These findings highlight the importance of targeted education to improve food management behaviors and reduce waste.

In Ethiopia, Wubetie et al. analyzed food insecurity using a geo-additive model and household survey data from 2012–2016. The study identifies northern and south-western Ethiopia as food insecurity hotspots, while the southeast and northwest are more food secure. Factors such as education, employment, urbanization, and agricultural practices reduce food insecurity, while natural disasters, small land ownership, and unemployment worsen it. The study highlights the role of rural vs. urban living, early marriage, and household size. The authors recommend targeted interventions in high-risk areas and further research on spatial effects and non-linear covariates in conflict-affected regions.

In Chile, Kanter et al. conducted a pilot study to assess a digital literacy intervention to strengthen local food systems. Targeting smallholder farmers, vendors, and consumers, the study sought to improve information and communication technologies (ICT) use amidst crises like the 2019 social unrest, COVID-19, and climate change. A 5-week intervention successfully boosted digital literacy, particularly among farmers and vendors. The study found a shift in food purchasing behavior, with more reliance on delivery services and social media-driven access during the pandemic. The research highlights the importance of digital literacy in enhancing food system resilience and recommends expanding such programs to other regions.

In Canada, Taylor et al. examine the ethical challenges facing Canadian grocers amid rising food prices and accusations of profiteering. Using consumer, watchdog, and industry data, the study highlights shifting public perceptions toward grocery chains. The authors argue that transparency and mandatory codes of conduct could restore trust while paradoxically resulting in the continued growth of corporate profit when societal needs are no longer perceived as being neglected or exploited. The analysis underscores the need for improved corporate accountability and transparent financial reporting to balance profit-making with public good.

Overall, the articles included in the Research Topic provide valuable insights into the sustainability and resilience of food systems in times of crises and shocks. They not only shed light on how crises and shocks, with a particular focus on the COVID-19 pandemic, impact food systems in terms of activities, functions, and outcomes but also highlight different practices as well as management and governance arrangements contribute to the transition toward sustainable and resilient food systems. Further research is needed to elucidate the sustainability-resilience nexus in food systems better and identify pathways to operationalize their functional linkages to prevent and/or mitigate the impacts of future crises and shocks.

